# Numerical simulation of autoclaved aerated concrete masonry wall subjected to close-in explosion and the structural damage assessment

**DOI:** 10.1038/s41598-024-54690-w

**Published:** 2024-02-16

**Authors:** Sheng Liu, Xiangyun Xu, Yilun Zhang, Bukui Zhou, Kezhi Yang

**Affiliations:** 1 Institute of Defense Engineering, AMS, PLA, Beijing, China; 2 Chinese People’s Liberation Army 31005 Unit, Beijing, China

**Keywords:** Autoclaved aerated concrete, Close-in explosion, Numerical simulation, Damage criterion, Structural materials, Civil engineering

## Abstract

In this study, we investigated the destructive effect of autoclaved aerated concrete (AAC) masonry walls subjected to close-in explosions. First, full-size refined finite-element models of the AAC masonry wall were established, and the accuracy of the models was verified by comparison with the test results. The destruction pattern and damage characteristics of the AAC wall were studied, and the effects of block size, wall thickness, mortar compressive strength, and explosion distance on the destruction degree of the AAC masonry walls were analyzed. The results showed that the destruction pattern of the AAC masonry wall subjected to close-in explosion manifested as punching damage in the middle of the wall. When the scaled distance remained unchanged, the punching damage area of the AAC masonry wall was positively correlated with the block size and negatively correlated with the wall thickness and mortar compressive strength. When the explosive equivalent remained unchanged and the explosion distance increased, the punching damage area first increased and then decreased. According to the damage mechanism of the AAC masonry wall, a calculation method for predicting the punching damage area of the AAC masonry wall was established, and the accuracy of this method was verified by comparing it with the numerical results. In addition, the damage criterion based on the punching damage area was established to determine the destruction levels of AAC masonry walls.

## Introduction

With the increasing frequency of terrorist attacks and accidental building explosions in recent years, the explosion resistance of important economic facilities and regular civil building structures has become a potential requirement and challenge among the engineering and academic communities^1–5^. As a significant component of the building, the masonry wall mainly functions as an enclosure and space separation. The masonry wall, which consists of an anisotropic layered structure composed of low-strength blocks and mortar, will experience serious damage due to its brittle nature and low integrity under explosion load, with the resulting debris and leakage pressure inevitably causing casualties and injuries for occupants and severe damage to equipment inside the building^6,7^. Therefore, there is an urgent need to study the dynamic response and damage effect of masonry walls under explosion load, to provide blast protection against various hazards.

Researchers worldwide have studied the damage effect analysis of masonry walls under explosion load. Ahmad et al.^8^ carried out six tests at large-scale distances to study the dynamic response of clay brick masonry walls under explosion load, while Knock et al.^9^ performed a near-field explosion test of clay brick masonry to investigate the distribution law of wall debris under explosion load and obtained an empirical formula to predict the velocity of debris based on fitting of the experimental data. Shi et al.^10^ conducted two near-field explosion tests of clay brick masonry walls to analyze the damage mechanism and crushing characteristics, and the results revealed that the size distribution of the fine portion of the fragments followed a Weibull distribution. Li et al.^11^ carried out experiments to assess the damage degree of clay brick masonry walls under gas explosion, and the results showed that with an increase in the wall thickness or decrease in wall height, the maximum displacement and damage level of the masonry walls decreased significantly. With significant improvements in computing power in recent years, numerical simulation technology has been widely used to investigate the explosion response of masonry walls. Shamim et al.^12–15^ carried out a series of studies on the dynamic response of different types of masonry walls under explosion load using numerical methods. The results showed that the explosion equivalent, standoff distance, detonation height, and detonation angle significantly influenced the damage level of the masonry walls. Furthermore, numerical algorithms to describe the damage characteristics of masonry walls^16–20^ and scaled numerical modeling methods^21–23^ have been developed to improve the numerical simulation accuracy and computational efficiency of unreinforced masonry walls under explosion load.

As a novel mortar-based material, autoclaved aerated concrete (AAC) has been extensively used in the masonry walls of various buildings with the features of excellent construction characteristics, a low mass-strength ratio, good heat insulation, and energy absorption properties. With the differences in raw materials and production processes, the mechanical properties of AAC blocks significantly differ from that of traditional clay bricks, attracting widespread attention from researchers worldwide^24,25^. Guo et al.^26^ performed a series of drop weight tests to assess the dynamic strength and absorption characteristics of AAC. The study showed that the stress–strain curve of AAC consisted of three stages under the low-velocity impact, with a slight effect of loading rate on the dynamic strength. Feng et al.^27^ conducted dynamic compressive strength tests on AAC with different densities in the strain rate range of 60–250 s^−1^ using a split Hopkinson pressure bar (SHPB) system. The results indicated an obvious enhancement in the dynamic compressive strength of AAC with an increase in density. Nian et al.^28^ assessed the dynamic response of AAC under an explosion shock wave generated by a shock tube, and the results revealed a significant influence of the pore structure and subsequent densification of AAC in the nonlinear stress strain relationship.

Due to the potential threat of conventional weapons and accidental explosions, explosion resistance and dynamic response under the explosion load of masonry walls have received significant attention. Yankelevsky and Avnon^29^ conducted a series of experiments on the local response of AAC under contact explosion, focusing on the reinforcement effect of polyvinyl acetate (PVA)-bonded woven fabric on AAC. The results showed that the tensile strength and ductility of AAC were effectively improved by the PVA-bonded woven fabric, which enhanced the tensile cracking resistance of AAC. Li et al.^30,31^ performed tests and numerical simulations to assess the influence of block strength, boundary conditions, and thickness on the explosion resistance of AAC masonry walls under gas explosions. According to the results, the influence of thickness and boundary conditions on the dynamic response of AAC walls was significant, while the influence of AAC strength on explosion resistance was minimal. Yu et al.^32^ conducted tests to investigate the failure mode of AAC masonry walls under far-range explosions, and explosion damage assessment criteria of the AAC walls were established based on the failure patterns of the specimens. Somayeh et al.^33^ analyzed the explosion resistance of AAC masonry walls under far-range explosions using numerical methods, and the effects of wall thickness and scaled distance on the dynamic response of the walls were determined.

Studies on the explosion resistance of masonry walls have mainly focused on far-field explosions with a standoff distance greater than two meters under contact explosion. Due to the different distributions of explosion load on the surfaces of masonry walls, these research results could not accurately indicate the dynamic response and damage of masonry walls under near-field explosions with a standoff distance of less than two meters. Moreover, research on the effecting factors of explosion resistance of AAC masonry walls, especially the effectiveness degree of block size and mechanical properties of masonry materials, has not been comprehensive. Finally, the existing damage assessment methods and failure criteria of masonry walls have been mainly applied to clay brick masonry walls. Due to the differences in mechanical properties of block materials and the different failure mechanisms of walls under explosion loads, these assessment criteria have not been able to accurately evaluate the explosion resistance of AAC masonry walls.

In this study, we investigated the nonlinear dynamic response and damage characteristics of AAC masonry walls subjected to a close-in explosion. First, full-size finite-element models of AAC masonry walls were established, and the CONWEP algorithm was used to apply explosion load to the finite-element models, with the accuracy of the models verified by comparison with the test results. Subsequently, the destruction pattern and damage characteristics of the AAC masonry walls subjected to close-in explosion were studied. The effects of block size, wall thickness, mortar compressive strength, and explosion distance on the destruction degree of the AAC masonry walls were then analyzed by the quantitative method. According to the damage mechanism of the AAC masonry walls, the calculation method for predicting the punching damage area of AAC masonry walls was established through theoretical analysis, and the accuracy of this method was verified by comparison with the numerical results. The damage criterion, based on the punching damage area, was also established to determine the destruction levels of AAC masonry walls.

## Numerical model verification

**Figure 1:**
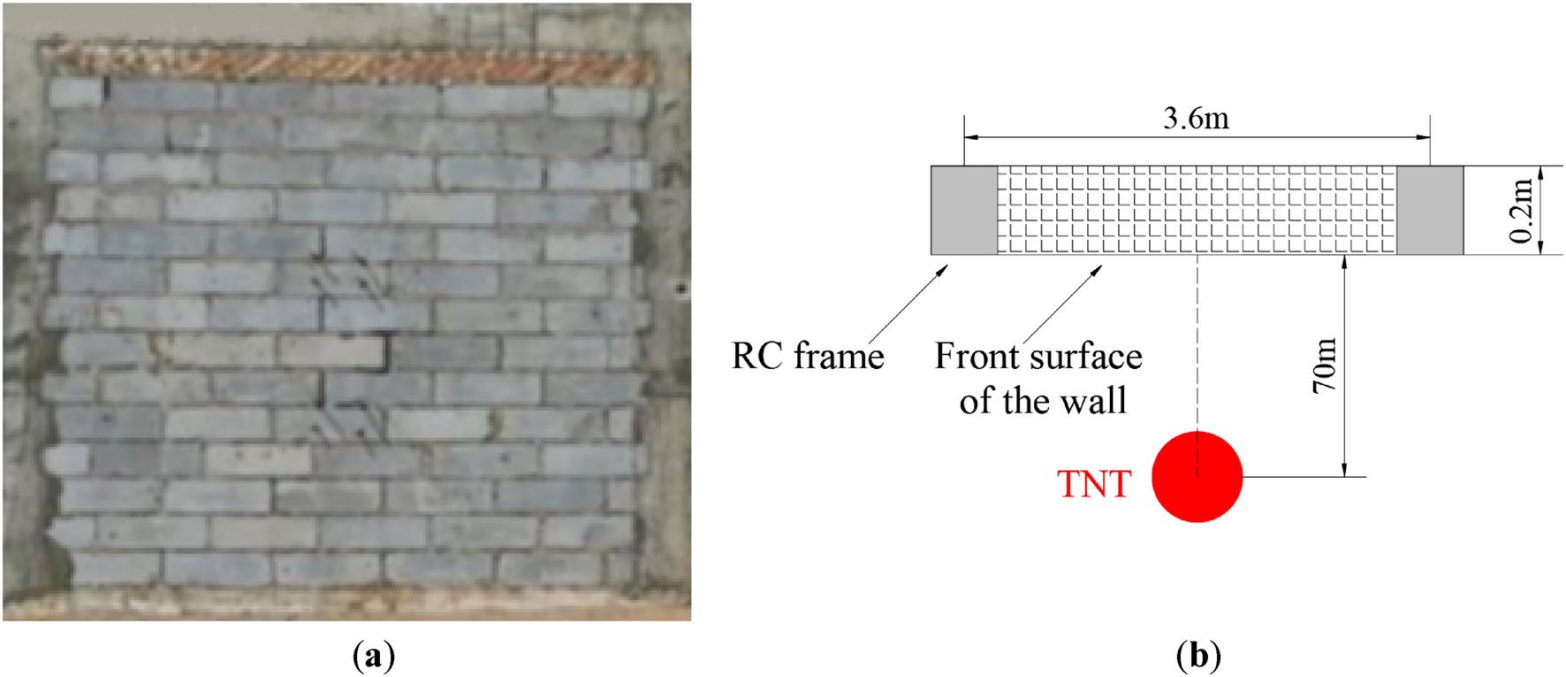
(**a**) AAC wall specimen^14^; (**b**) test set-up.

Due to fast propagation speed, high peak pressure, and short-duration characteristics, the dynamic response and failure modes of AAC masonry blocks will significantly differ from those under static load. Yu et al.^32^ conducted dynamic response and damage tests of an AAC masonry wall under explosion load, where during the test, 3000 kg of TNT charge was placed 70 m in front of the AAC masonry wall. The AAC masonry wall specimens with dimensions of 3.6 × 3.6 × 0.2 m, and the experimental setup, are shown in Fig. 1. The basic mechanical parameters of the AAC were as follows: the uniaxial compressive strength was 3.5 × 10^6^ Pa, Young’s modulus was 5.3 × 10^8^ Pa, the Poisson’s ratio was 0.2, the mean density was 500 kg/m^3^, and the dimensions of the block were 0.6 × 0.6 × 0.2 m. The mortar had a compressive strength of 7.6 × 10^6^ Pa. Subsequently, the dynamic response and damage of the AAC masonry wall under explosion load were simulated to verify the reliability and accuracy of the numerical model.

### Finite element model

**Figure 2:**
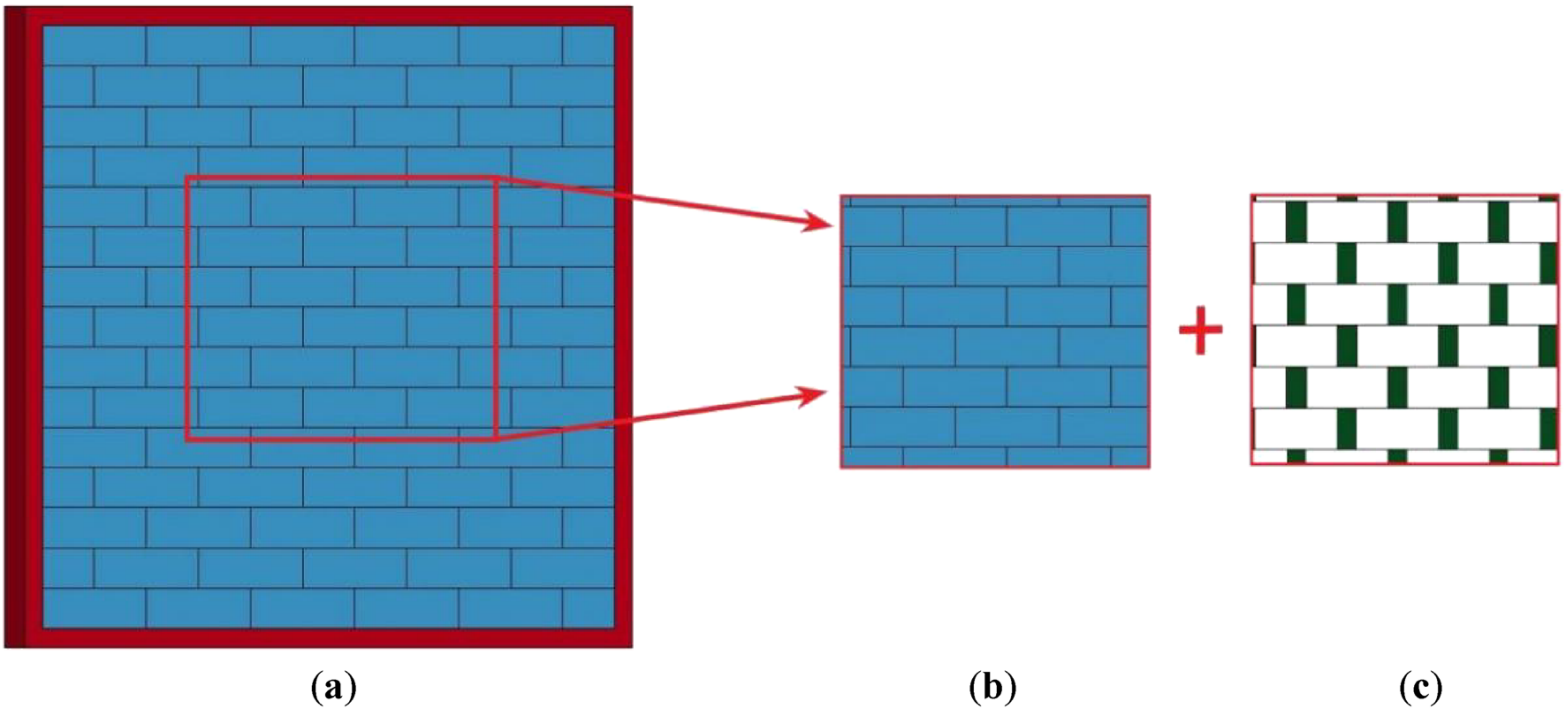
(**a**) The entire model; (**b**) block model; (**c**) mortar model.

The numerical model of the AAC masonry wall was divided into a separation model and a homogenization model. The separation model was established by treating the block and mortar separately, to accurately simulate the bonding slip and failure processes between the blocks and mortar. Due to the small difference in mechanical properties between the AAC and mortar, the contact surface with zero-thickness could be applied to replace the mortar. In this section, a separation model of the AAC masonry wall was developed using commercial finite element software LS-DYNA, as shown in Fig. 2. The AAC block and external frame adopted the Lagrangian calculation method, in which the coordinates moved with the material, where all node displacement and rotation of the concrete frame were fixed.

### Material model

The material model *Mat_96 (MAT_BRITITLE_DAMAGE) was employed to model the AAC blocks. This model consisted of an anisotropic brittle damage model, which could be applied to a wide variety of brittle materials. According to the GB/T11971-1997 test standard^34^, Li et al.^30^ poured 0.1 m AAC cube specimens and used an MTS servo-hydraulic testing machine to assess the mechanical properties of the AAC specimen and determine the material model parameters of the AAC block. The tensile strength, shear strength, fracture energy, and shear retention factor were obtained from Ref.^30^, while the density, compressive yield strength, modulus of elasticity, and Poisson’s ratio were obtained from Ref.^32^. The detailed model parameter values are listed in Table 1. To accurately simulate AAC masonry wall failure under explosion load and prevent the excessive distortion of elements, erosion criteria were used to simulate the failure of materials. The maximum principal strain was used as the failure criterion, and the value was defined as 0.01. Of note, the value of failure strain lacked a relevant theoretical basis, which was determined mainly by trial calculations in this study.

**Table 1:** Material parameters for the AAC blocks.

Density (kg/m^3^)	Young’s modulus (Pa)	Poisson’s ratio	Tensile strength (Pa)	Shear strength (Pa)	Compressive yield strength (Pa)	Fracture energy (N/m)	Shear retention factor
500	6.3 × 10^8^	0.2	7 × 10^5^	1 × 10^6^	3 × 10^6^	80	0.03

Due to the high strength and stiffness of the external frame, minimal damage occurred to the external frame in previous tests. Moreover, the research object of this numerical model was the damage behavior and dynamic response. Therefore, a Rigid model was applied to model the external frame.

### Method of load application

The fluid–solid coupling method and CONWEP method have been commonly used to apply explosion load. The fluid–solid coupling method could establish the air domain and calculate the propagation process of the explosion shock wave in air. The CONWEP method simplified these processes, which effectively reduced the complexity of the finite element model and improved the calculation efficiency. Therefore, the CONWEP method was employed to apply explosion loads with different TNT mass values and different explosion distances to the AAC masonry wall.

CONWEP, an explosion load calculation method developed on the basis of fitting a large number of experimental data^35^, was built using the LS-DYNA program, and the explosion load was applied to the selected surface by combining the *LOAD_BLAST_ENHANCED and *LOAD_BLAST_SEGMENT keywords. The overpressure time curve could be represented by adopting the modified Friedlander equation, according to

1 $$ P_{{\text{S}}} \left( t \right) = P_{{{\text{S}}0}} \left( {1 - \frac{t}{{t_{0} }}} \right)e^{{ - b\frac{t}{{t_{0} }}}} , $$

where *P*_*S*0_ is the peak incident overpressure, *t*_0_ is the positive phase duration, and *b* is the decay attenuation coefficient of the waveform. The peak overpressure on the structure was also related to the incident angle θ. The CONWEP method could generate the equivalent overpressure value, as expressed by

2 $$ P\left( t \right) = P_{ref} \left( t \right) \cdot \cos^{2} \theta + P_{in} \left( t \right) \cdot \left( {1 + \cos^{2} \theta - 2\cos \theta } \right), $$

where *P*_*ref*_ is the reflected pressure and *P*_*in*_ is the incident pressure, which could be calculated by Eq. (1).

### Contact surface

The bonding between the AAC blocks as well as between the AAC wall and external frame was simulated as tie-break contacts in the numerical model, to accurately describe separation and sliding between the contact surfaces. Contact was achieved using the *TIEBREAK_SURFACE_TO_ SURFACE command in LS-DYNA^36^. This contact was governed by the stress-based failure criterion, according to

3 $$ \left( {\frac{{f_{n} }}{{F_{n} }}} \right)^{2} + \left( {\frac{{f_{s} }}{{F_{s} }}} \right)^{2} > 1, $$

where *f*_*n*_ and *f*_*s*_ denote the normal stress and shear stress on the contact surface, respectively, and *F*_*n*_ and *F*_*s*_ are the normal failure stress and shear failure stress, respectively, which could be characterized by the tensile strength and shear strength of the mortar. When the stress state on the contact surface satisfied Eq. (3), mutual contact movement between the blocks would be constrained by friction. In this study, the static and dynamic coefficients of friction factor were 0.4 and 0.3, respectively, while the normal failure stress *F*_*n*_ and shear failure stress *F*_*s*_ were 3 × 10^5^ Pa. The above parameters were obtained from the trial calculation and validated by the experimental data.

### Mesh size

In the numerical simulations, which simulated interactions between the explosion load and masonry wall, the accuracy of the numerical results depended significantly on the mesh size of the finite element model. To ensure the accuracy of the numerical results, convergence analysis of the finite element model mesh size was carried out to determine the appropriate parameters in this section.

**Figure 3:**
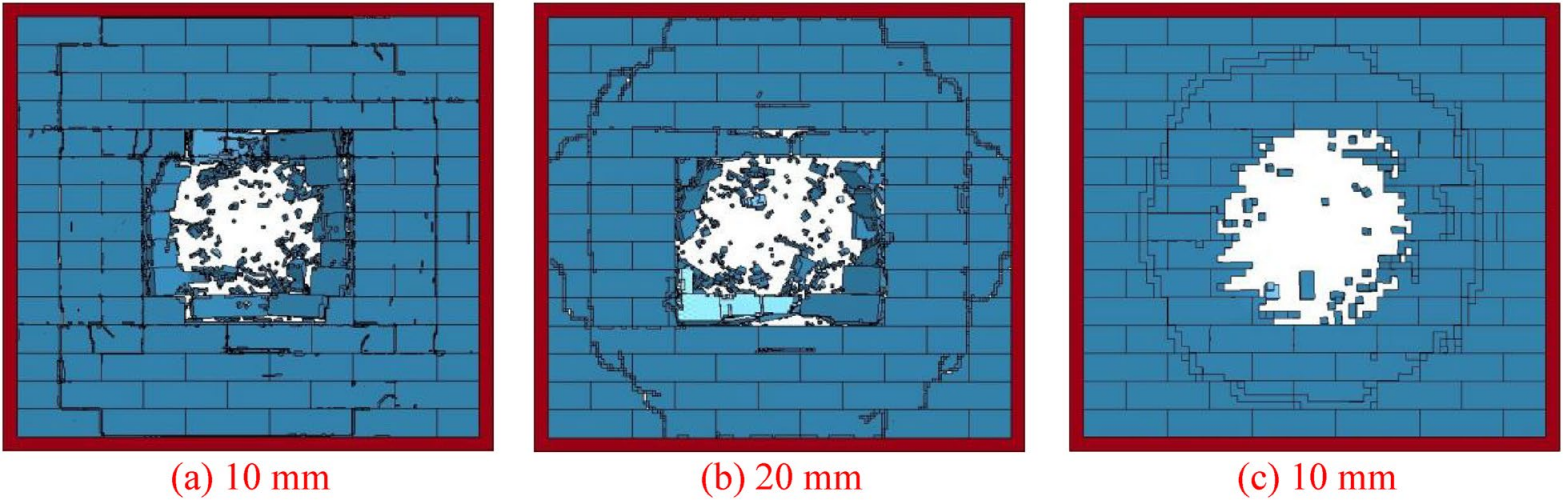
The final damage determined by finite element models with different mesh sizes.

Three finite element models of the AAC masonry wall were built with mesh sizes of 0.01, 0.02, and 0.05 m to simulate interactions between the explosion load and the AAC masonry wall. Figure 3 illustrates the final damage of the finite element models with different mesh sizes when the explosive equivalent was 1 kg and the explosion distance was 0.5 m. With a decrease in mesh size, the final damage of the AAC masonry wall tended to converge. However, we observed that the finer the solid mesh, the more accurate the calculation of the final AAC masonry wall damage. Considering the calculation accuracy and computational efficiency, a mesh size of 20 mm was chosen, and the finite element model had a total of 280,000 solid elements, 353,265 nodes, and 1,485,000 degrees of freedom.

### Comparison of the experimental and numerical results

**Figure 4:**
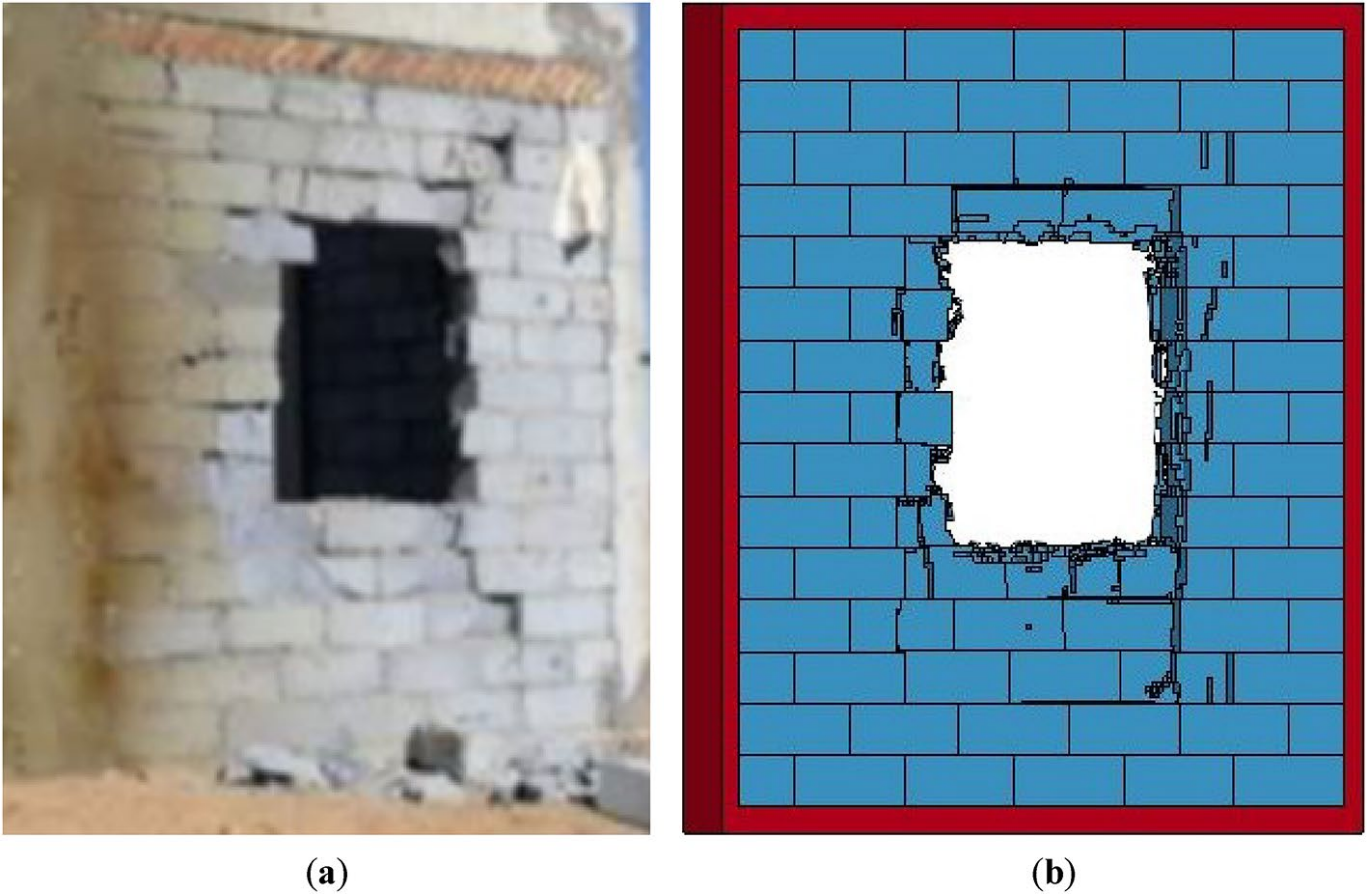
(**a**) Experimental results^32^; (**b**) numerical results.

The final damage of the AAC masonry wall in the test is shown in Fig. 3. The surfaces of certain blocks in the margin of the AAC masonry wall developed fragmentation and peeling, and a 1.2 × 1.2 m square hole developed in the center portion of the AAC masonry wall. Relatively intact AAC blocks fell off the front and back of the wall, which was mainly caused by the destruction of the cement mortar. Figure 4 shows the final damage of the AAC masonry wall in the numerical calculation. Due to the failure of elements and the displacement of the blocks after the failure of the contact surface, a 1.3 × 1.2 m rectangular hole developed in the center portion of the wall. The failure mode and damage area of the AAC masonry wall in the numerical calculation essentially agreed with the experimental results, which verified the accuracy of the numerical model and the material parameters.

## Dynamic response of the AAC masonry wall under close-in explosion

In this section, the dynamic response and damage failure of the AAC masonry wall under close-in explosion load were numerically simulated. Based on the simulation results, the dynamic response characteristics and damage failure modes of the AAC masonry wall under close-in explosion load were analyzed. Moreover, the influence of parameters such as the block size, wall thickness, mortar compressive strength, and explosion standoff distance on the dynamic response and damage of the AAC masonry wall under close-in explosion was assessed.

### Typical simulation results

**Figure 5:**
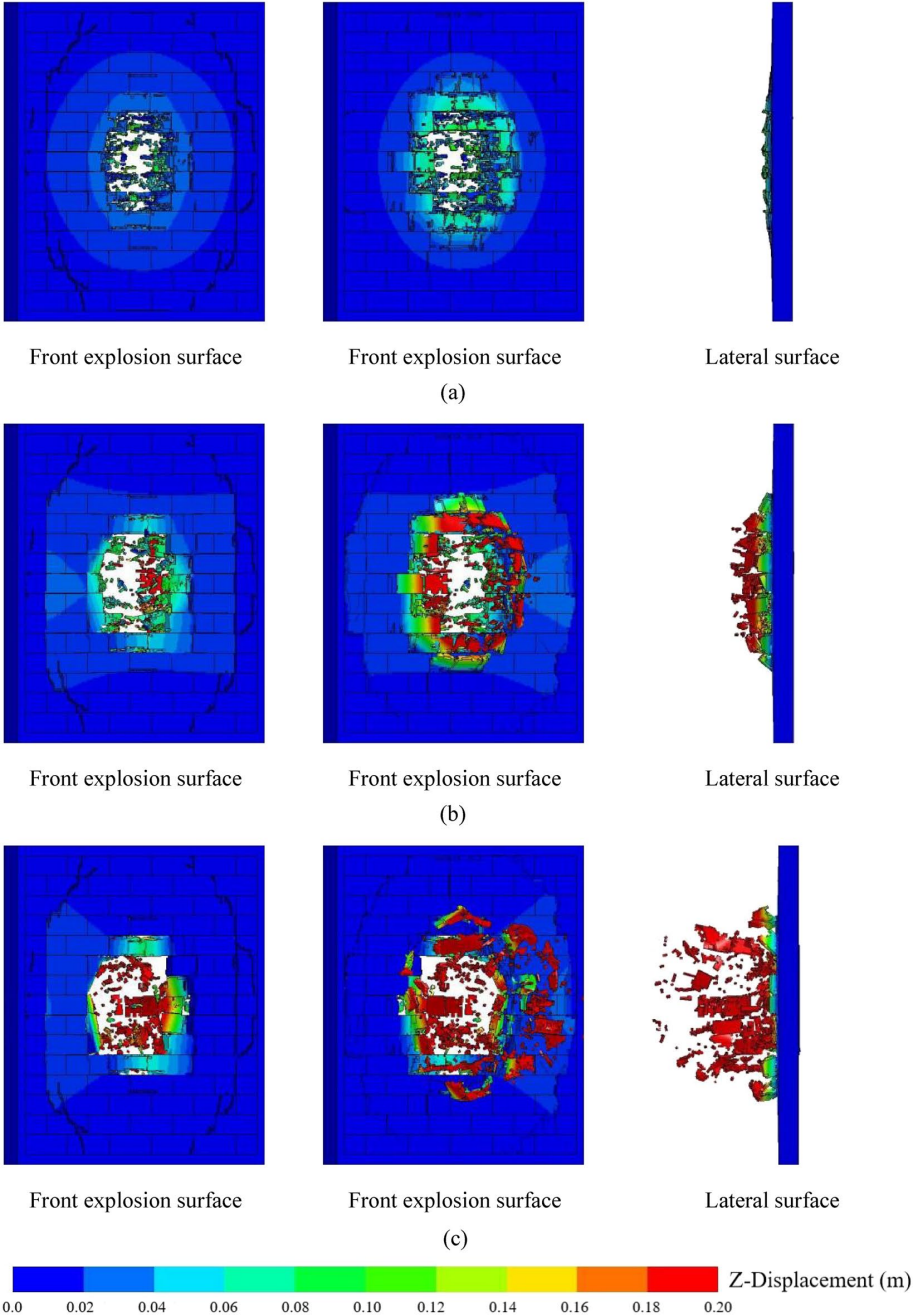
(**a**) Displaced cloud of the AAC masonry wall when *t* = 0.01 s; (**b**) displaced cloud of the AAC masonry wall when *t* = 0.05 s; (**c**) displaced cloud of the AAC masonry wall when *t* = 0.2 s.

An AAC masonry wall with a width of 3.3 m, height of 3.0 m, and thickness of 0.2 m was selected as the typical model. The dimensions of the AAC blocks were 600 × 200 × 200 mm, with a compressive strength of 3.5 × 10^6^ Pa. Figure 5 shows a series of displacement isosurfaces at different times, including block displacement at three moments, clearly showing the destruction and movement of the blocks. At *t* = 0.01 s, the energy generated by the explosion was absorbed by the wall and propagated in the form of stress waves in the wall. Serious punching failure was observed in the middle portion of the wall. Slight compression failure occurred in the margin portion of the front explosion surface, and peeling failure occurred on the back explosion surface under the action of the tensile stress wave. At *t* = 0.05 ms, damage to the AAC masonry wall was further aggravated, and the stress of the contact surface between certain blocks in the margin portion exceeded the shear limit, causing the movement of blocks away from the wall. At *t* = 0.2 s, the punching failure portion of the AAC masonry wall further expanded, and a rectangular hole formed in the middle portion of the wall due to damage and slip of the blocks. According to the numerical results, the contact surface between the blocks was the vulnerable portion under close-in explosion load, which was prone to damage, and wall damage occurred in the form of punching failure.

### Parameter study

The AAC masonry wall was composed of blocks and mortar, and the explosion resistance of the wall was affected by the sizes of the blocks and the mechanical properties of the mortar. In addition, for the same mass of TNT charge, the pressure distribution on the surface of the wall varied with a change in explosion distance. In this section, the effects of block sizes, wall thickness, mortar compressive strength, and explosion distance on the dynamic response and damage degree of the AAC masonry wall under close-in explosion were discussed.

#### Effect of block size

To explore the differences in AAC masonry wall explosion resistance with different block sizes, six finite element models with different block sizes were constructed. The mesh size and material parameters were consistent with Section "Dynamic response of the AAC masonry wall under close-in explosion". In the calculation examples, the distances from the TNT charge to the center of the wall were all 0.5 m, and the explosive equivalent was 1 kg. Table 2 shows the peeling area and punching hole area of the AAC masonry wall. When the number of masonry blocks was increased from 60 to 255, the peeling failure area of the front explosion wall surface decreased by 20.7%, and the punching hole area decreased by 8.8%. The reason was that when the dimensions of the wall were the same and the size of the blocks decreased, the amount of mortar used for masonry bonding increased, and the explosion energy absorbed by the mortar also increased. Due to the higher strength of the mortar than the blocks, the damage degree of the AAC masonry wall was reduced.

**Table 2:** Damage to the AAC walls with different block sizes under close-in explosion.

Block size (m)	Number of blocks	Contact area between the blocks and mortar (m^2^)	Peeling failure area (m^2^)	Punching hole area (m^2^)
0.60 × 0.30 × 0.20	60	20.4	8.28	1.71
0.60 × 0.20 × 0.20	90	27.0	7.84	1.77
0.60 × 0.15 × 0.20	120	33.6	7.02	1.70
0.40 × 0.20 × 0.20	135	30.6	6.65	1.64
0.20 × 0.20 × 0.20	255	40.2	6.56	1.56

**Figure 6:**
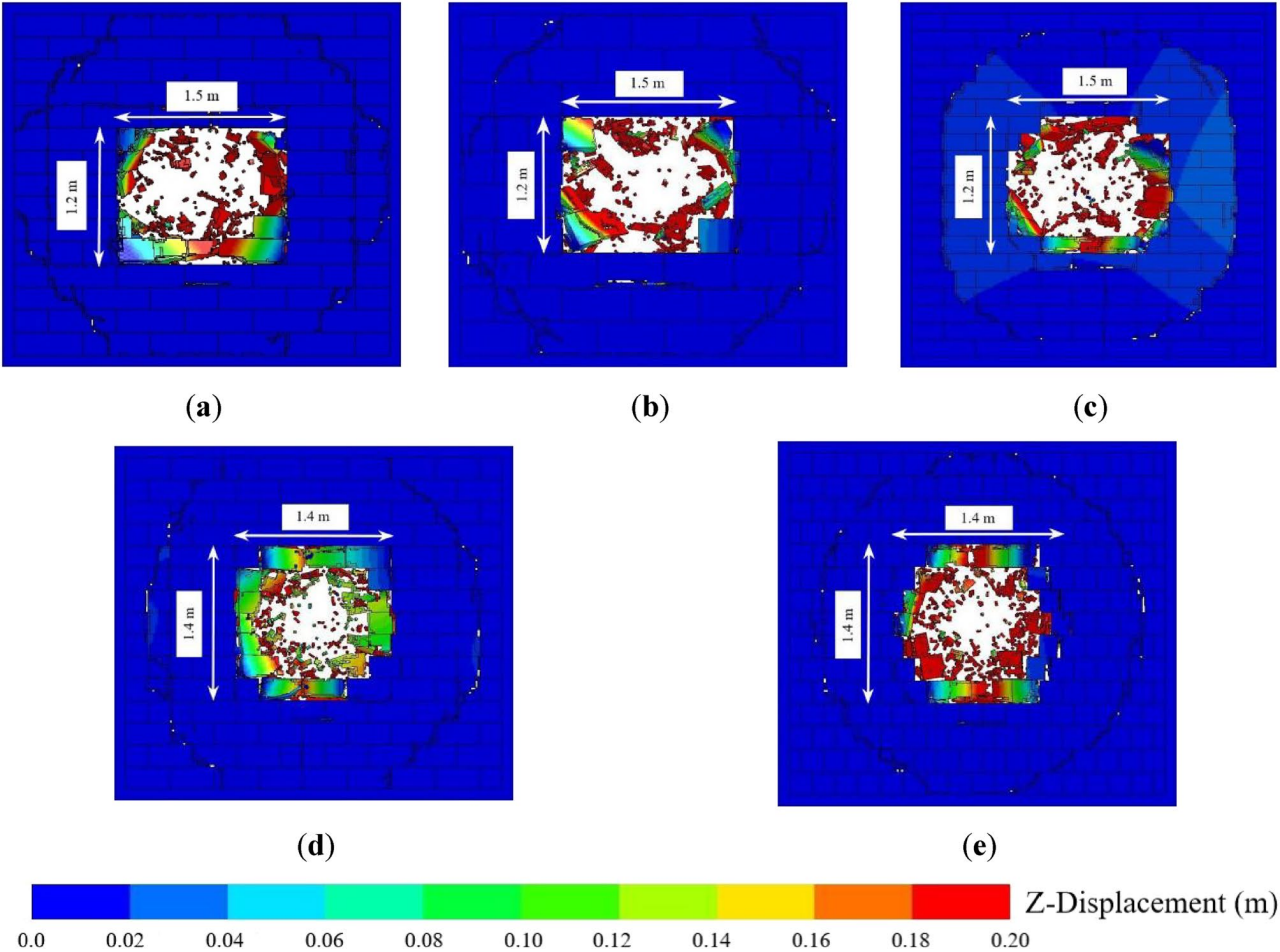
(**a**) Final damage of the AAC wall with a block size of 0.60 × 0.20 × 0.20 m; (**b**) final damage of the AAC wall with a block size of 0.60 × 0.30 × 0.20 m; (**c**) final damage of the AAC wall with a block size of 0.60 × 0.15 × 0.20 m; (**d**) final damage of the AAC wall with a block size of 0.40 × 0.20 × 0.20 m; (**e**) final damage of the AAC wall with a block size of 0.20 × 0.20 × 0.20 m.

Figure 6 shows the final damage to the AAC walls with different block sizes under close-in explosion, revealing that the peeling area and punching hole of the AAC wall were almost symmetrically distributed around the center of the wall. When the length of the block was constant, the transverse size of the punching hole was the same, and the lateral size was an integer multiple of the block height. When the height of the block was constant, the lateral size of the punching hole was the same, and the transverse size of the punching hole in the masonry wall with a small block size and identical length and height was an integer multiple of the block length. In addition, the transverse size of the punching hole for the masonry wall with a large block size and aspect ratio greater than one was a half-integer multiple of the block length. The reason for this phenomenon was that when the block size was small with a larger lateral stiffness and longitudinal stiffness, the blocks were not prone to punching failure. When the height of the blocks was small and the length was large, the lateral stiffness was lower than the longitudinal stiffness, and shear failure would occur in the middle of the blocks. As the vulnerable portion of the AAC masonry wall, the bonding layer between the blocks was damaged first under close-in explosion load, which caused the movement of the blocks. Therefore, the lateral size of the AAC masonry wall punching hole was an integer multiple or half-integer multiple of the length of the blocks, and the longitudinal size was an integer multiple of the block height.

In summary, with a decrease in block size, the shear resistance effect of the block increased, and the area of the punching hole of the AAC masonry wall decreased, while the shape of the punching hole changed from a rectangle to an approximate circle. However, the numerical results also showed that the decrease in block size had a limited effect on the improved explosion resistance of the AAC masonry wall.

#### Effect of the wall thickness

The dynamic responses and damage effects of the AAC masonry walls with different thicknesses under the same explosion differed. To explore the influence of thickness on the AAC masonry wall dynamic response and damage effect under explosion load, five numerical models of the AAC masonry wall were built with thicknesses of 100, 150, 200, 250, and 300 mm. The grid division and material parameters were consistent with Section "Numerical model verification". The distances from the TNT charge and wall were 0.5 and 2.0 m, respectively, and the explosive equivalent was 1 kg.

Table 3 summarizes the final damage condition of the AAC masonry wall with different thicknesses. When the explosion distance was 0.5 m, punching failure occurred in the AAC masonry walls with different thicknesses. With an increase in wall thickness from 0.1 to 0.3 m, the punching hole area decreased by 90.6%. When the explosion distance was 2.0 m, the middle area of the masonry wall with a thickness of 0.1 m was slightly damaged, and the dynamic response of the other walls was within the elastic range. With an increase in wall thickness from 0.1 to 0.3 m, the maximum displacement of the wall decreased by 99.6%. The reason for this phenomenon was that with an increase in wall thickness, the sectional inertial moment parallel to the direction of explosion loading and bonding force between the mortar and blocks increased. Therefore, the shear capacity of the masonry wall significantly improved, which enhanced its explosion resistance.

**Table 3:** Final damage condition of the AAC masonry wall with different thicknesses.

Explosive equivalent (kg)	Explosion distance (m)	Thickness of wall (mm)	Final damage condition
1	0.5	0.10	Punching damage in the overall area, where the punching hole area was 8.31 m^2^
		0.15	Punching damage in the overall area, where the punching hole area was 7.82 m^2^
		0.20	Punching damage in the middle area, where the punching hole area was 1.96 m^2^
		0.25	Punching damage in the middle area, where the punching hole area was 0.94 m^2^
		0.30	Punching damage in the middle area, where the punching hole area was 0.78 m^2^
1	2.0	0.10	Slight damage, where the maximum displacement was 2.3 × 10^−2^ m
		0.15	Elastic deformation, where the maximum displacement was 8.7 × 10^−4^ m
		0.20	Elastic deformation, where the maximum displacement was 4.2 × 10^−4^ m
		0.25	Elastic deformation, where the maximum displacement was 1.7 × 10^−4^ m
		0.30	Elastic deformation, where the maximum displacement was 8.0 × 10^−5^ m

**Figure 7:**
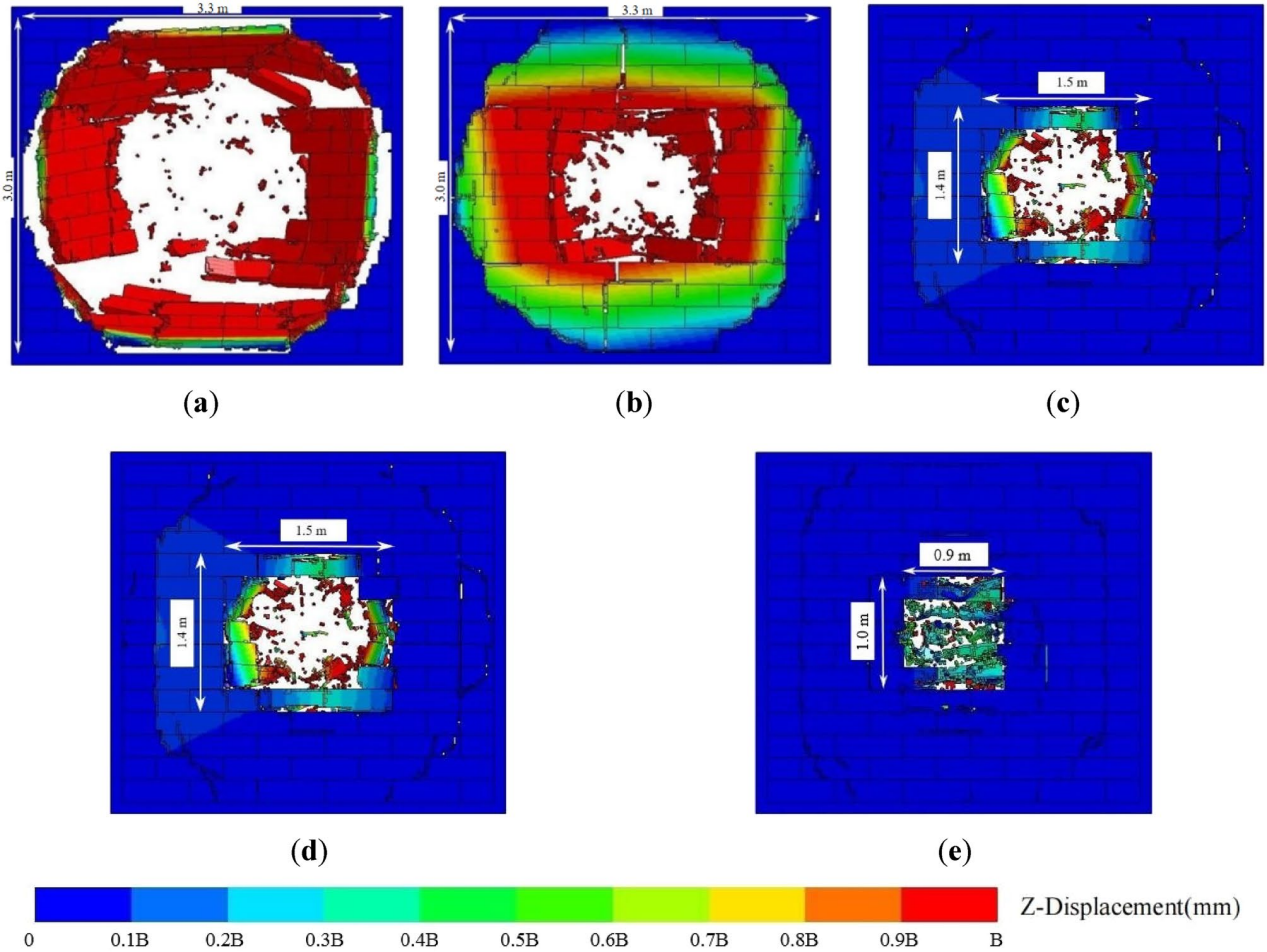
(**a**) Final damage of the AAC wall with a thickness of 0.10 m; (**b**) final damage of the AAC wall with a thickness of 0.15 m; (**c**) final damage of the AAC wall with a thickness of 0.20 m; (**d**) final damage of the AAC wall with a thickness of 0.25 m; (**e**) final damage of the AAC wall with a thickness of 0.30 m.

Figure 7 shows the final damage condition of masonry walls with different thicknesses when the explosion distance was 0.5 m, with B denoting the thickness of the masonry wall. When the thickness of the wall was 0.10 m, the blocks in the middle area of the masonry wall were crushed because the pressure exceeded the strength of the material, and the surrounding area was ejected from the frame in an integral form, and the punching failure area was approximately circular. When the wall thickness was increased to 0.15 m, the punching failure area was relatively reduced, and its shape remained approximately circular. With a further increase in wall thickness, only slight peeling damage occurred in the surrounding area of the masonry wall. The range of crushed blocks in the middle area of the masonry wall gradually decreased, and the shape of the punching hole changed from circular to square. The reason for this phenomenon was that when the wall thickness was small, the shear resistance of the masonry wall was low, and the block was prone to punching failure with a shape distribution of the explosion load. Therefore, the punching hole was approximately circular. With an increase in thickness, the sectional shear resistance of the masonry wall was enhanced, and the middle of the block and contact surfaces between the block and mortar were vulnerable. As a result, shear failure occurred when the explosion loading was high. Therefore, the punching hole was approximately square.

#### Effect of mortar compressive strength

The compressive strength of the mortar served as a key factor that influenced the explosion resistance of the AAC masonry wall. To analyze the influence of mortar compressive strength on the damage degree of the wall under close-in explosion, four numerical AAC masonry wall models were built with mortar compressive strengths of 2.5, 5, 7.5 and 10 MPa. The TNT explosion quantity was 1 kg, and the explosion distances were 0.5 and 0.2 m, where the corresponding scaled distances were 0.5 and 2 m/kg^1/3^. In this section, mortar with different compressive strengths was obtained by changing the normal failure stress and shear failure stress values in the TIEBREAK contact. Equations (4) and (5) present the calculation methods of the average tensile strength σ_t_ and average shear strength σ_v_ of the brick masonry along the straight joint, according to Chinese code GB55007-2021^34^:

4 $$ \sigma_{t} = 0.125\sqrt {f_{c} } , $$

5 $$ \sigma_{v} = 0.125\sqrt {f_{c} } , $$

where *f*_*c*_ is the compressive strength of mortar.

**Figure 8:**
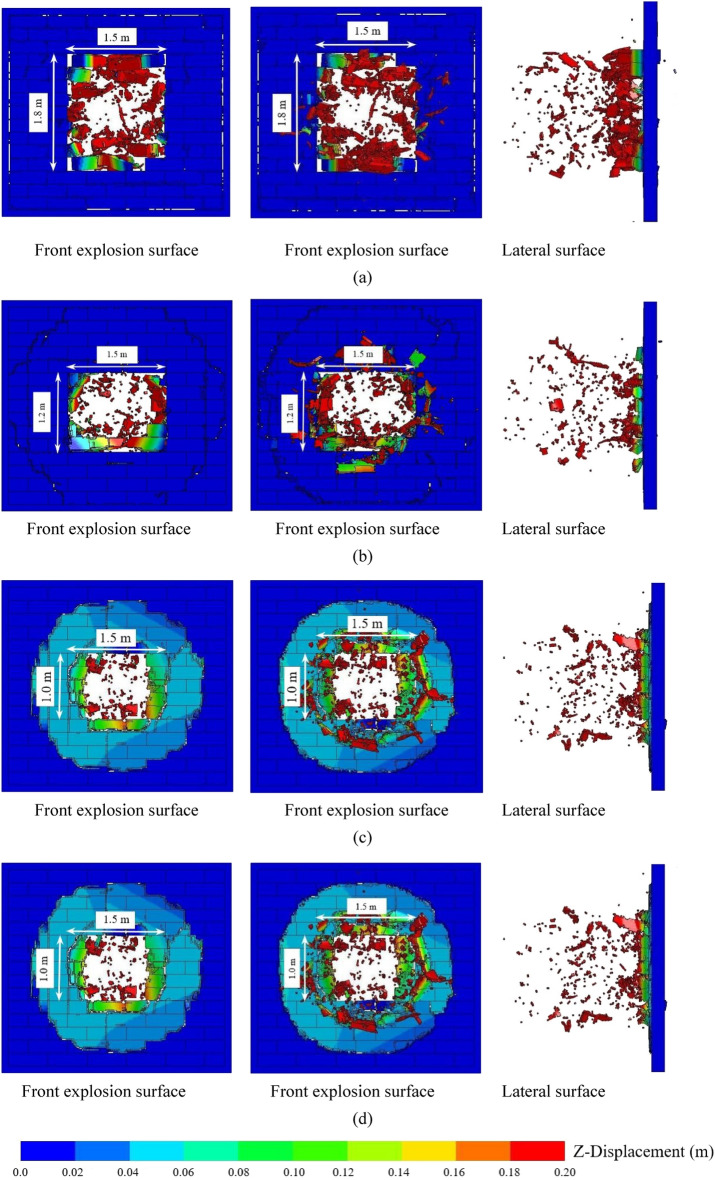
(**a**) Mortar compressive strength of 2.5 × 10^6^ Pa; (**b**) mortar compressive strength of 5.0 × 10^6^ Pa; (**c**) mortar compressive strength of 7.5 × 10^6^ Pa; (**d**) mortar compressive strength of 1.0 × 10^6^ Pa.

Figure 8 shows the final damage of the AAC masonry wall with different mortar compressive strengths when the scaled distance was 0.5 m/kg^1/3^. When the compressive strength was 2.5 × 10^6^ Pa, partial bonding between the wall and frame was damaged, and the margin portion of the front and back explosion surfaces of the wall developed serious peeling. In addition, a punching hole appeared in the middle area of the wall. When the compressive strength was 5.0 × 10^6^ Pa, no failure cracks appeared at the bonds between the wall and the frame. The peeling areas of the front and back explosion surfaces of the wall decreased by 12.1%, and the punching hole area was reduced by 23.0%. When the compressive strength was increased to 1.0 × 10^7^ Pa, the peeling areas of the front and back wall explosion surfaces decreased by 46.4%; thus, the punching hole area was reduced by 65.5%. This indicated that the damage degree of the AAC masonry wall decreased with an increase in mortar compressive strength.

**Figure 9:**
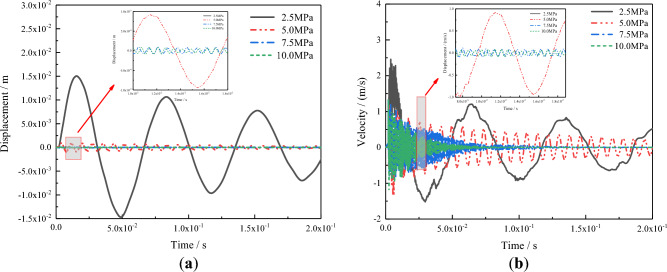
(**a**) Displacement time history curve; (**b**) velocity time history curve.

Figure 9 shows a comparison between the different mortar compressive strengths of the center displacements and velocity time history curves of the AAC masonry wall when the scaled distance was 2 m/kg^1/3^. Under this explosion load, the dynamic response of the AAC masonry wall was in the elastic range. With an increase in mortar compressive strength, the displacement and velocity peak of the wall center gradually decreased, and the vibration frequency gradually increased, while the time for the wall to return to the static range gradually decreased.

The above analysis showed that with an increase in the compressive strength of the mortar, the bonding surface strength between the block and mortar increased, which induced an increase in the integrity of the AAC masonry wall and enhanced the dissipation effect of the wall on the explosion energy. The maximum displacement and damage degree of the masonry wall tended to decrease. Therefore, enhancing the compressive strength of the mortar allowed the wall to improve the structural resistance to explosion damage.

#### Effect of explosion distance

With the same explosive equivalent, the pressure and impulse distribution on the surface of the wall changed with a variation in explosion distance. To investigate the influence of explosion distance on the damage degree of the AAC masonry wall, nine different explosion distance numerical models were built, and the explosive quantity was 2 kg. Table 4 summarizes the final damage condition of the AAC masonry walls with different explosion distances. With an increase in explosion distance, the punching hole area of the wall first increased and then decreased. In these calculation examples, the damaged area was concentrated in the middle portion of the masonry wall. With an increase in explosion distance, the punching failure area first increased and then decreased. When the ratio of explosion distance to charge radius exceeded 30, the explosion loading on the wall surface significantly decreased, which was less than the bonding force between the mortar and the blocks. Only the central area slightly exceeded the compressive strength of the AAC, which caused minor peeling on the surface of a few blocks. Therefore, with an increase in the explosion distance, the failure mode of the AAC masonry wall changed from severe punching failure to minor peeling failure.

**Table 4:** Damage degree condition of the AAC masonry wall with different explosion distances.

Explosive equivalent (kg)	Explosion distance (m)	Scaled distance (m/kg^1/3^)	R/R_W_	Final damage condition
2	0.50	0.397	7.53	Punching hole area of 3.48 m^2^
2	0.60	0.476	9.03	Punching hole area of 3.90 m^2^
2	0.70	0.556	10.54	Punching hole area of 3.96 m^2^
2	0.80	0.635	12.05	Punching hole area of 4.23 m^2^
2	0.90	0.714	13.55	Punching hole area of 4.14 m^2^
2	1.00	0.794	15.06	Punching hole area of 3.58 m^2^
2	1.25	0.992	18.82	Punching hole area of 2.58 m^2^
2	1.50	1.191	22.59	Punching hole area of 2.06 m^2^
2	2.00	1.587	30.11	Peeling area of 0.53 m^2^

**Figure 10:**
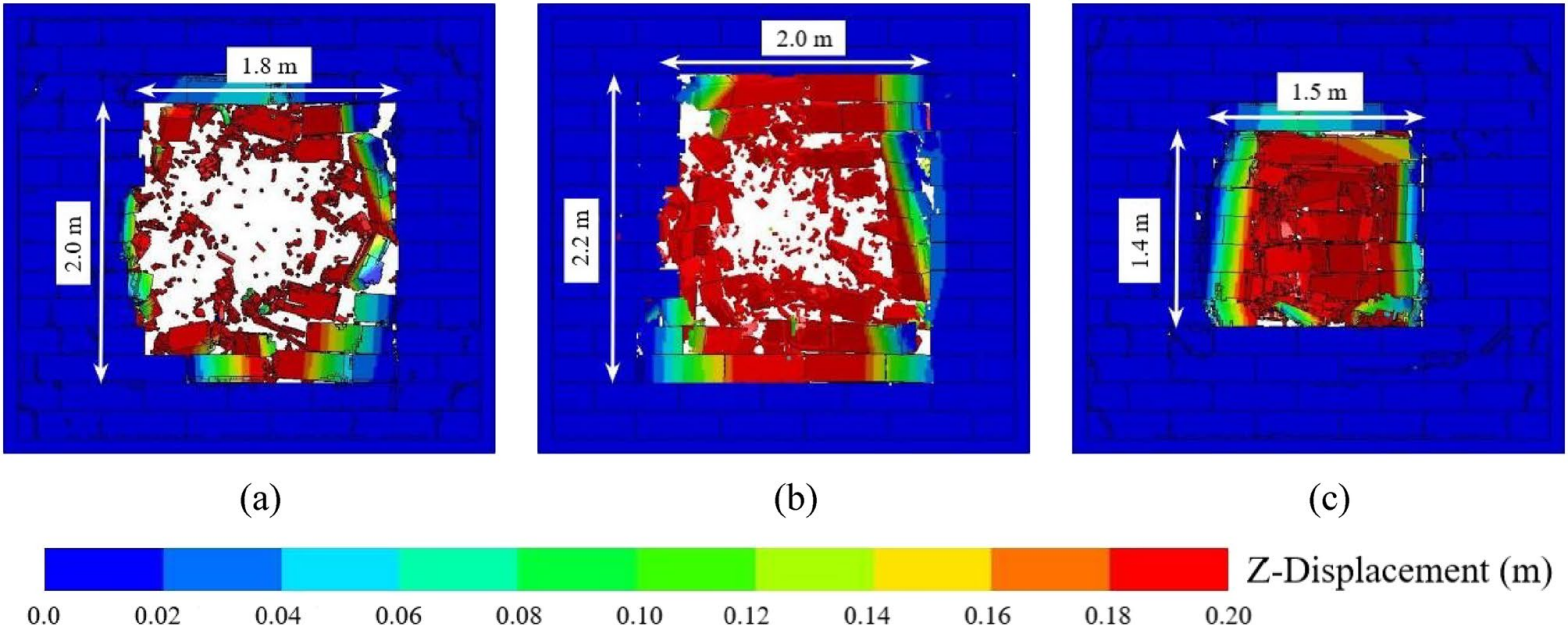
(**a**) Final damage of the AAC masonry wall when the explosion distance was 0.5 m; (**b**) final damage of the AAC masonry wall when the explosion distance was 0.8 m; (**c**) final damage of the AAC masonry wall when the explosion distance was 1.5 m.

Figure 10 shows the final damage of the AAC masonry walls when the explosion distances were 0.5, 0.8, and 1.5 m. When the explosion distance was 0.5 m, a large number of fragments were generated due to the high explosion load in the middle portion of the wall, which exceeded the compressive strength of the block. The weak explosion load in the margin portion of the wall did not cause the destruction of the blocks; however, some blocks were displaced due to the failure of the contact surfaces, thus forming a punching hole. When the explosion distance was increased to 0.8 m, the explosion load in the middle portion of the wall was relatively reduced and the number of fragments relatively decreased. However, the number of blocks that fell off the wall increased, which caused an increase in the area of the punching hole. When the explosion distance was further increased to 1.5 m, few fragments were observed in the middle area of the wall, and the area of blocks that fell off the wall was relatively reduced, which led to a decrease in the area of the punching hole. The reason for this phenomenon was that when the explosion distance was small, the energy generated by the explosion mainly acted on the middle area of the wall. The material strength of the blocks played a major role in resisting the explosion, and the bond strength between the blocks played a minor role. As the explosion distance increased, the explosion energy acting on the wall gradually decreased, and the explosion load distributed on the wall became more homogeneous. The proportion of explosion energy dissipated by the material strength of the blocks gradually decreased, and the proportion of explosion energy dissipated by the bonding strength between the blocks gradually increased. Therefore, the area of compressed blocks gradually decreased, and the range of blocks that fell off the wall gradually increased, which caused an increase in the punching hole area of the wall. With a further increase in the explosion, the bonding strength between the blocks played a main role in resisting the explosion load. Due to the reduced explosion energy on the wall, the range of blocks that fell off the wall gradually decreased, and the punching hole area of the AAC masonry wall decreased.

**Figure 11:**
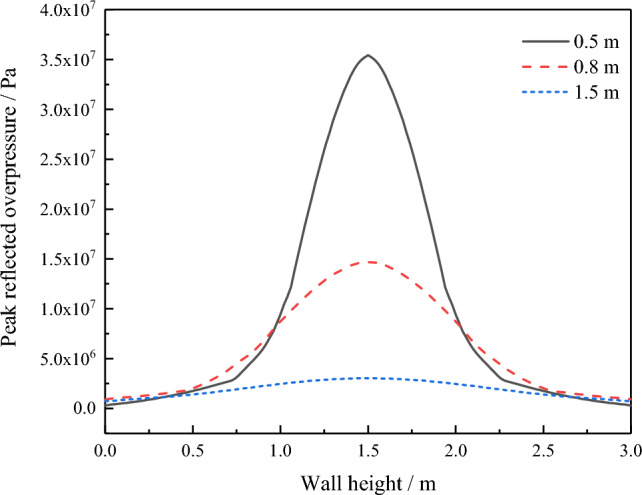
Distribution of peak value of the reflected overpressure along the height of the wall with different explosion distances.

Figure 11 shows the distribution of the peak value of shock wave reflection overpressure along the wall height under the above three conditions. The results revealed that the overpressure in the middle area of the wall gradually decreased with an increase in explosion distance, and the load concentration of the wall gradually increased, which caused a load range expansion of the contact surface between the blocks. Therefore, the area of blocks that fell off the wall increased. With a further increase in explosion distance, the explosion load on the wall decreased and showed a more uniform distribution. The explosion load range that caused the failure of the contact surface between blocks was reduced, and the damage degree of the wall decreased. This further showed that when the explosive equivalent was constant, the damage degree of the AAC masonry wall first increased and then decreased with an increase in explosion distances.

## Damage assessment

The reasonable definition of failure criterion is an important prerequisite for the evaluation of damage degree of the structural members. Commonly used failure criteria of masonry walls involve scaled distance, the ultimate displacement of the wall, and the rotation angle of the support. However, these failure criteria can only assess whether the masonry wall will be damaged and cannot assess the damage degree of the masonry wall. In this section, we proposed a damage criterion based on the punching failure area of the wall, which was used to assess the damage degree of the masonry wall under close-in explosion load.

### Calculation method of the punching hole area in the masonry wall

The damage mechanism of masonry walls under close-in explosion load was different from that of a homogeneous structure. To analyze the punching hole area of the masonry wall under close-in explosion load, the following assumptions were proposed. (1) The explosive was placed in front of the center of the front surface of the wall to ensure that the incident angle was zero degrees when the explosion shock wave propagated and impacted the front surface of the wall. (2) The time when the explosion shock wave reached each point of the wall was neglected. (3) Friction between the mortar and blocks was neglected.

**Figure 12:**
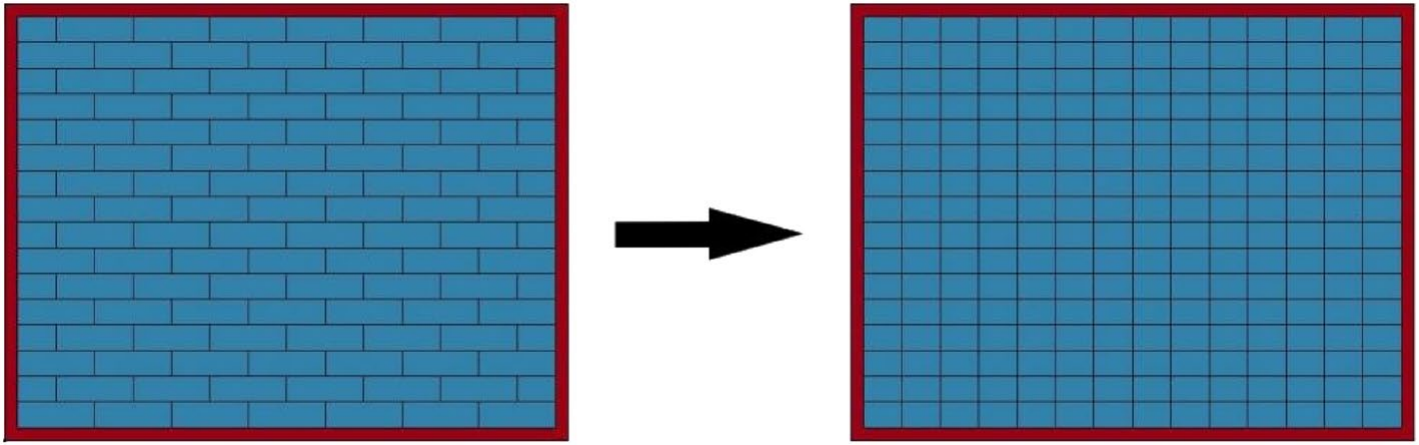
Analysis model of the masonry wall.

As previously mentioned, the transverse size of the punching hole of the masonry wall under close-in explosion load was the integer multiple or half-integer multiple of the length of the block, and the longitudinal size consisted of an integer multiple of the block height. To simplify this analysis, a zero-thickness bonding surface was inserted in the middle of the block to transform a complete block into two half-blocks, as shown in Fig. 12.

**Figure 13:**
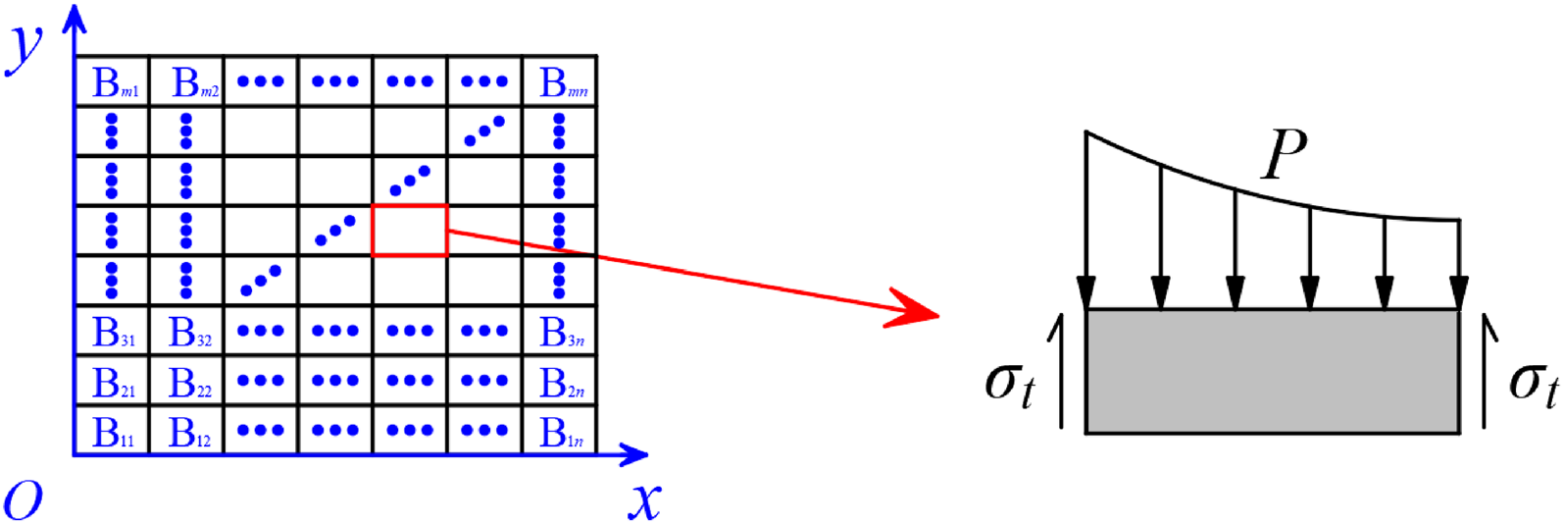
The punching failure analysis model of the masonry wall.

Due to the load and structure symmetry, the 1/4 structure was analyzed. Under close-in explosion load, the blocks in the middle area of the masonry wall were crushed due to high pressure, and the blocks in the margin area fell off the wall due to failure of the bonding surface between the mortar and blocks. Therefore, the punching hole area of the masonry wall was mainly determined by the bond strength between the mortar and blocks. A coordinate system was established with the center of the masonry wall as the origin, and the blocks were numbered and analyzed, as shown in Fig. 13. The bonding force *I* and explosion load *F* of the block were determined by

6 $$ I = \iint {\sigma_{t} d}S_{c} = 2\sigma_{t} \left( {lb + bh} \right), $$

7 $$ F = \iint {P\left( r \right)dS_{p} }, $$

where *σ*_*t*_ is the bonding strength between mortar and block, *S*_*c*_ is the contact surface between the mortar and block, *l*, *b*, and *h* denote the length, width, and height of the block, respectively, and *S*_*p*_ is the front explosion surface. When the explosive load of the block exceeded the bonding force, the blocks fell off the wall.

For a given set of working conditions, the following data were obtained: *m* (block rows), *n* (block columns), *l*_0_ (block length), *b*_0_ (block width), *h*_0_ (block height), *σ*_*t*0_ (bonding strength), and *P*_0_ (explosion load). We then substituted *l*_0_, *b*_0_, *h*_0_, and *σ*_*t*0_ into Eq. (6) to obtain *I*_*0*_ and substituted *P*_0_ into Eq. (7) to acquire *F*_0_. The process of determining the corner block *B*_*edge*_ at the punching hole was as follows:

1. If *I*_*mn*_ < *F*_*mn*_, the block *B*_*mn*_ was located at the corner of the punching hole.
2. If *I*_*mn*_ ≥ *F*_*mn*_, the relationship between *I*_*m*−1*n*−1_ and *F*_*m*−1*n*−1_ of block *B*_*m*−1*n*−1_ had to be compared.
3. If *I*_*m*−1*n*−1_ < *F*_*m*−1*n*−1_, the relationships between *I*_*mn*−1_ and *F*_*mn*−1_ of block *B*_*mn*−1_ and between *I*_*m*−1*n*_ and *F*_*m*−1*n*_ of block *B*_*m*−1*n*_ had to be compared.

If *I*_*mn*−1_ < *F*_*mn*−1_, *I*_*m*−1*n*_ ≥ *F*_*m*−1*n*_, then block *B*_*mn*−1_ was located at the corner of the punching hole.

If *I*_*mn*−1_ ≥ *F*_*mn*−1_, *I*_*m*−1*n*_ < *F*_*m*−1*n*_, then block *B*_*m*−1*n*_ was located at the corner of the punching hole.

If *I*_*mn*−1_ ≥ *F*_*mn*−1_, *I*_*m*−1*n*_ ≥ *F*_*m*−1*n*_, then block *B*_*m*−1*n*−1_ was located at the corner of the punching hole.

(4) If *I*_*m*−1*n*−1_ ≥ *F*_*m*−1*n*−1_, the relationship between *I*_*m*−2*n*−2_ and *F*_*m*−2*n*−2_ of block *B*_*m*−1*n*−1_ was compared, until the block at the corner of the punching hole was determined.

Assuming that the horizontal distance from the upper right corner of *B*_*edge*_ to the center of the wall was *L* and the longitudinal distance was *H*, the punching hole area *S* of the masonry wall could be obtained by

8 $$ S = 4LH. $$

Table 5 shows a comparison between the numerical and calculation results of the punching hole area of the masonry wall in Section "Dynamic response of the AAC masonry wall under close-in explosion", indicating that most of the calculation results were higher than the numerical results. This was because friction between the bond surface, mortar, and blocks was neglected in this assumption, and the constraint force of the block in the calculation model was less than the actual constrained force. As a result, the calculated punching failure area was higher than the numerical results. The error between the numerical results and calculation results did not exceed 15%, which met the actual engineering requirements. Therefore, the calculation method of the punching hole area of the masonry wall proposed in this section is effective.

**Table 5:** Comparison of numerical results and calculation results of punching failure area of AAC masonry wall.

Explosive equivalent (kg)	Explosion distance (m)	Numerical result (m^2^)	Calculating result (m^2^)	Error (%)
1	0.5	1.59	1.80	13.21
	1.0	0.56	0.54	− 3.57
	1.5	–	–	–
	2.0	–	–	–
2	0.5	3.48	3.60	3.45
	1.0	3.58	3.78	5.59
	1.5	2.06	2.10	1.94
	2.0	0.53	0.60	13.2

### Calculation method of punching failure area of masonry wall

According to the numerical results, the damage of AAC masonry wall could be divided into the following degrees: (a) low damage, where slight peeling damage occurred on the front and backs of the wall, and no blocks fell off the wall; (b) moderate damage, where serious peeling damage occurred on the front and backs of the wall, and a few relatively intact blocks fell off the wall; (c) high damage, where large number of blocks fell off the wall; (d) collapse damage, where the wall completely disintegrated and collapsed.

**Figure 14:**
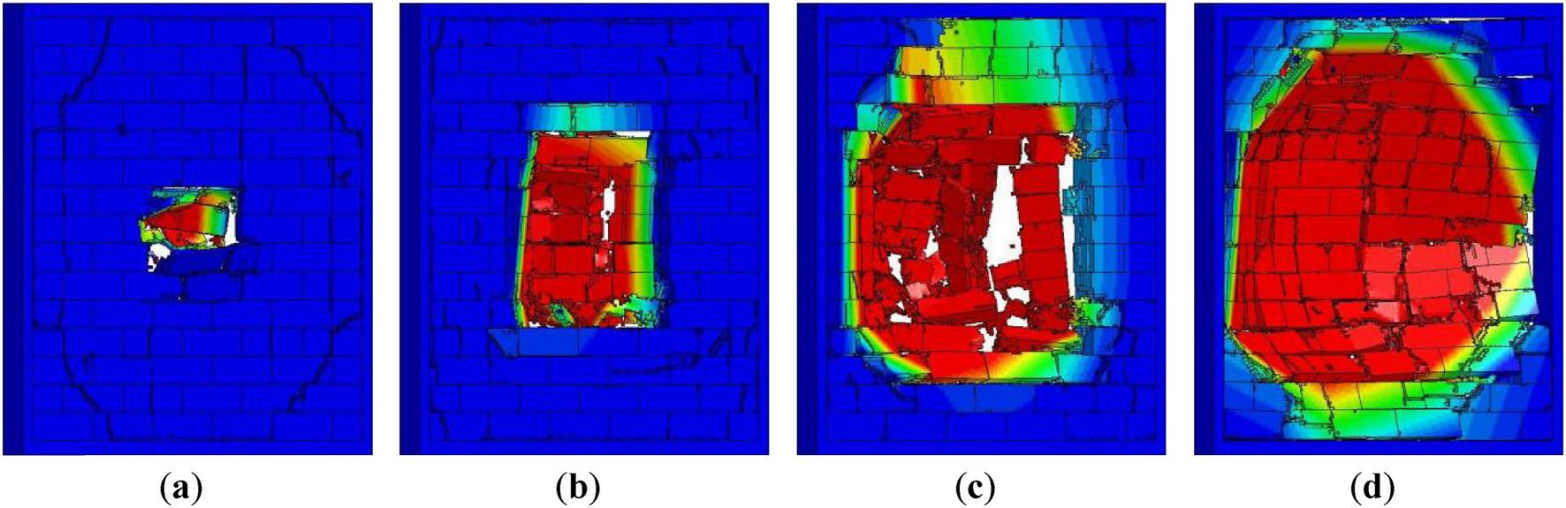
(**a**) Low damage; (**b**) moderate damage; (**c**) high damage; (**d**) collapse damage.

According to the numerical results, an empirical damage criterion for AAC masonry walls under close-in explosion could be obtained. The damage parameter D could be defined as

9 $$ D = \frac{{S_{W,perforation} }}{{S_{W} }} $$

where *S*_*w*_ is the perforation of the punching hole area, and *S*_*w*_ is the area of the wall surface. When 0 < *D* ≤ 0.2, the wall experienced low damage; when 0.2 < *D* ≤ 0.5, the wall experienced moderate damage; when 0.5 < *D* ≤ 0.8, the wall experienced high damage; when 0.8 < *D* ≤ 1, the wall experienced collapse damage. The final failure conditions of the AAC masonry walls with different damage degrees are shown in Fig. 14.

## Conclusions

In this study, we investigated the dynamic response process and failure characteristics of AAC masonry walls under the action of explosion loading. A full-size three-dimensional finite element model of AAC masonry wall was constructed, and the accuracy of the numerical model was verified by comparison with previous experimental results. In this study, the failure process and mode of the AAC masonry wall were analyzed. The effects of different block sizes, wall thicknesses, mortar compressive strengths, and explosion distances on the damage degree of the AAC masonry wall were discussed. According to the analytical results, key conclusions could be drawn, as follows.

1. The failure mode and damage degree of the AAC masonry wall numerical model were consistent with the experimental results, indicating that the proposed numerical method could accurately simulate the dynamic response and damage behavior of AAC masonry walls under explosion loading. The AAC masonry wall exhibited local punching damage under close-in explosion.
2. With a decrease in block size, the damage degree of the AAC masonry wall was reduced, and the horizontal stiffness of the block was more significant than the vertical stiffness. However, the effects of block size on the explosion resistance performance of AAC masonry walls were very limited.
3. With an increase in wall thickness, the moment of inertia of the section parallel to the explosion load direction and bonding force between the mortar and blocks increased, the shear resistance of the masonry wall significantly increased, the area of the punching hole relatively decreased, while the shape of the punching hole changed from circular to square. Increasing the wall thickness allowed the wall to reduce the damage degree of the AAC masonry wall under close-in explosion.
4. With an increase in mortar compressive strength, the bonding strength between the mortar and blocks increased, causing an improvement in the integrity of the AAC masonry wall and an increase in the dissipation effect of the wall on the explosion energy, as well as a decrease in the maximum displacement and damage degree of the AAC masonry. Enhancing the compressive strength of mortar allowed the wall to improve structural resistance to explosion damage.
5. When the explosive equivalent remained unchanged and the explosion distance increased, the dissipation subject of explosion energy transformed from the material strength of the block to the bonding strength between the mortar and blocks, where the damage degree of the AAC masonry walls first increased and then decreased. Therefore, the combined effects of explosive equivalent and explosion distance need to be fully considered when assessing the damage degree of AAC masonry walls.
6. A method for calculating the punching hole area of masonry walls under close-in explosion load was established, and the effectiveness of the method was verified by comparison with the numerical results. A damage criterion based on the punching hole area of the AAC masonry wall was proposed to assess the damage degree of the wall under close-in explosion load. This method could provide a theoretical basis for the study of explosive energy dissipation by AAC masonry walls in the future.

## Data Availability

The datasets used and/or analyzed during the current study are available from the corresponding author upon reasonable request.
